# Repurposing anticancer drugs for COVID-19-induced inflammation, immune dysfunction, and coagulopathy

**DOI:** 10.1038/s41416-020-0948-x

**Published:** 2020-06-22

**Authors:** Kamal S. Saini, Carlo Lanza, Marco Romano, Evandro de Azambuja, Javier Cortes, Begoña de las Heras, Javier de Castro, Monika Lamba Saini, Sibylle Loibl, Giuseppe Curigliano, Chris Twelves, Manuela Leone, Mrinal M. Patnaik

**Affiliations:** 1 Covance Inc, Princeton, NJ USA; 2 East Suffolk and North Essex NHS Foundation Trust, Ipswich, UK; 3 Institut Jules Bordet, Brussels, Belgium; 4 Université Libre de Bruxelles (U.L.B), Brussels, Belgium; 5 IOB Institute of Oncology, Quiron Group, Madrid, Spain; 6 Vall d’Hebron Institute of Oncology, Barcelona, Spain; 7 Medica Scientia Innovation Research, Ridgewood, NJ USA; 8 Madrid Medical Doctors Association, Madrid, Spain; 9 La Paz Hospital, Madrid, Spain; 10 HistoGeneX, Antwerp, Belgium; 11 GBG, Neu-Isenburg, Germany; 12 Centre for Haematology and Oncology Bethanien, Frankfurt, Germany; 13 Istituto Europeo di Oncologia, IRCCS, Milan, Italy; 14 University of Milano, Milan, Italy; 15 University of Leeds and Leeds Teaching Hospitals Trust, Leeds, UK; 16 Mayo Clinic, Rochester, MN USA

**Keywords:** Drug development, Viral infection, Drug development

## Abstract

Three cardinal manifestations of neoplasia, namely inflammation, immune dysfunction, and coagulopathy are also seen in patients with severe SARS-CoV-2 infection, providing a biological rationale for testing selected anticancer drugs for their ability to control the symptoms and/or modify the course of COVID-19.

## Main

The coronavirus disease 2019 (COVID-19) pandemic, caused by the severe acute respiratory syndrome coronavirus-2 (SARS-CoV-2), has resulted in >5.9 million infections and 363,000 deaths as of 29 May 2020.

Although SARS-CoV-2 primarily infects the upper and lower respiratory tract, it can also affect the intestine, heart, liver, kidney, brain, and other organs.^1^ Among 1482 patients with confirmed COVID-19 in the United States, the most common signs and symptoms included cough (86%), fever or chills (85%), shortness of breath (80%), diarrhoea (26%), and nausea or vomiting (24%).^2^

No treatment has shown convincing benefit yet for patients with COVID-19, but the Food and Drug Administration (FDA) recently granted emergency use authorisation for the repurposed investigational anti-Ebola drug remdesivir for COVID-19. Repurposing refers to the use of approved or investigational drugs beyond the scope of the original medical indication.^3^ Repurposing, not only of antiviral drugs but also those used in other diseases such as cancer, is worthy of consideration to shorten timelines for identifying an effective therapy for COVID-19.

### Inflammation, immune dysfunction, and coagulopathy in COVID-19 and cancer

Although many of the details regarding the SARS-CoV-2 virus and its effects on humans are yet to be elucidated, a few interesting commonalities between the pathophysiology of COVID-19 and cancer are beginning to emerge. Notably, both these diseases exhibit the triad of inflammation, immune dysregulation, and coagulopathy.

The intracellular entry of SARS-CoV-2 is facilitated by the angiotensin-converting enzyme 2 receptor, which is expressed in type II alveolar cells of lung, cholangiocytes, oesophageal keratinocytes, ileal and colonic enterocytes, myocardial cells, renal proximal tubule cells, bladder urothelial cells, fibroblasts, endothelial cells, oral mucosal epithelium, and haematopoietic cells, including monocytes and macrophages.^4^ Crosstalk between monocytes, macrophages, and other antigen-presenting cells could explain some features of inflammation and immune dysfunction in severe COVID-19.

Viral infections may trigger host inflammation resulting in the production of cytokines that lead to vasodilation, neutrophil extravasation, and leakage of plasma into the infected tissue.^5^ Intracellular multiplication of SARS-CoV-2 results in increased levels of the pro-inflammatory interleukin (IL)-6, tumour necrosis factor (TNF)-α, IL-1β, IL-2, interferon (IFN)-γ, and IL-10.^6^ There are three main pathways of IL-6 signal transduction: *cis* signalling, *trans* signalling, and *trans* presentation. In classic, *cis*-mediated signalling IL-6 binds to its membrane-bound receptor mIL-6R that is present on immune cells and modulates Janus kinases (JAKs) and signal transducer and activator of transcription 3 (STAT3); in *trans*-mediated signalling IL-6 binds to its soluble receptor sIL-6R, potentially impacting all cell surfaces; while in *trans*-mediated presentation, IL-6 binds to mIL-6R on immune cells leading to downstream T cell signalling precipitating acute respiratory distress syndrome.^7^ IL-6 inhibitors are known to suppress *cis* and *trans* signalling but not *trans* presentation.

Dysregulated immune responses in critically ill patients with COVID-19 is reflected by lymphopenia, affecting mostly CD4^+^ T cells, including effector, memory, and regulatory T cells, and decreased IFN-γ expression in CD4^+^ T cells.^8^ Exhaustion of cytotoxic T lymphocytes, activation of macrophages, and a low human leukocyte antigen-DR expression on CD14 monocytes has been noted in patients with COVID-19.

A marked pro-coagulant tendency has been observed in patients with severe COVID-19^9^ and may present as microvascular or macrovascular thrombosis affecting the lung, heart, intestine, kidney, or other organs, with elevated D-dimer, fibrin/fibrinogen degradation products, fibrinogen level, or disseminated intravascular coagulation.^10^ Out of 184 patients admitted with COVID-19, 31% had thrombotic complications despite standard thromboprophylaxis, with pulmonary embolism being the most common event.^11^ A multifactorial process termed as microvascular COVID-19 lung vessel obstructive thromboinflammatory syndrome could play a role in the rapid evolution of multiorgan injury.^12^

Important manifestations of severe COVID-19 infection are shared with neoplasia, namely inflammation, immune dysfunction, and coagulopathy. Inflammation has been long known to play a central role in cancer pathogenesis, and in 2011, Hanahan and Weinberg labelled tumour-promoting inflammation as a hallmark of cancer. Chronic inflammation is both a risk factor and a consequence of cancer. Innate cytotoxic cells as well as the adaptive immune cells are dysfunctional in cancer, allowing neoplastic cells to avoid detection and elimination by the immune system. Thromboembolism is recognised as a leading cause of death in patients with cancer, with the risk of venous thrombosis increased several fold.^13^

### Repurposing anticancer drugs against COVID-19

The clinical development of a new drug or vaccine usually takes several years. Given the urgent need to quickly find efficacious therapies for COVID-19, existing drugs are being repurposed and tested in clinical trials, potentially substantially accelerating development timelines. The pharmaceutical industry, contract research organisations (CROs), and academia have spent decades developing drugs for cancer-induced inflammation, immune dysfunction, and coagulopathy; given that this triad is also seen in patients affected by COVID-19, it is reasonable to consider testing selected anticancer agents in a rational manner against this viral illness.

Several drugs that have been approved for a cancer indication by the US FDA are now in COVID-19 clinical trials (see Table 1). These include the anti-interleukin tocilizumab, which competitively blocks the IL-6-binding site and is approved for managing the cytokine release syndrome that is often observed in patients treated with chimeric antigen receptor (CAR) T cells and bispecific antibodies; siltuximab, which prevents the binding of IL-6 to both soluble and membrane-bound IL-6 receptors and is approved for multicentric Castleman’s disease; corticosteroids like prednisolone and dexamethasone, which are used in lymphomas and leukaemias; enoxaparin used for the prophylaxis of deep vein thrombosis in patients with cancer; bevacizumab, which binds vascular endothelial growth factor and is approved for several solid cancers; immunomodulators like thalidomide and lenalidomide used for multiple myeloma; IFN-α used for hairy cell leukaemia, myeloproliferative neoplasms, melanoma, and follicular lymphoma; checkpoint inhibitors like the programmed death receptor-1 inhibitors nivolumab and pembrolizumab that are approved for several types of cancers; tyrosine kinase inhibitors like imatinib, duvelisib, and acalabrutinib; antimetabolites; topoisomerase II inhibitors; and even radiotherapy. In addition, CAR therapy, approved for some haematological cancers, is also being studied in COVID-19 (clinicaltrials.gov identifier NCT04324996). Finally, there are several drugs and cell and gene therapies in clinical development for a cancer indication that are now being tested for efficacy against COVID-19.

**Table 1:** Approved anticancer agents being tested in patients with COVID-19.

Class	Agent	Mechanism	US FDA approval for cancer type or cancer symptom	COVID-19 trial identifier
Interleukin (IL) inhibitor	Tocilizumab	Competitive blockade of the IL-6-binding site	Cytokine release syndrome	NCT04361552, NCT04331795
	Siltuximab	Prevents the binding of IL-6 to both soluble and membrane-bound IL-6 receptors	Multicentric Castleman’s disease	NCT04329650, NCT04330638
Corticosteroid	Prednisolone	Anti-inflammatory and immunosuppressive	Lymphomas, leukaemias	NCT04273321, NCT04263402
	Dexamethasone	Anti-inflammatory and immunosuppressive	Lymphomas, leukaemias	NCT04325061, NCT04327401
	Hydrocortisone	Anti-inflammatory and immunosuppressive	Palliation of leukaemias and lymphomas	NCT04348305, NCT02735707
Anticoagulant	Enoxaparin	Binds to antithrombin to irreversibly inactivate clotting factor Xa	Prophylaxis of deep vein thrombosis in abdominal surgery or medical patients with severely restricted mobility during acute illness	NCT04345848, NCT04359277
Interferon	IFN-α	Immunomodulator	Hairy cell leukaemia, melanoma, follicular lymphoma	NCT04320238, NCT04254874
Checkpoint inhibitor	Nivolumab	Blocks programmed death-1 receptor	Melanoma, non-small cell lung cancer, renal cell cancer, Hodgkin’s lymphoma, squamous cell cancer of the head and neck, urothelial cancer, colorectal cancer, hepatocellular cancer	NCT04333914, NCT04356508
	Pembrolizumab	Blocks programmed death-1 receptor	Melanoma, non-small cell lung cancer, head and neck squamous cell cancer, Hodgkin’s lymphoma, primary mediastinal large B-cell lymphoma, urothelial cancer, microsatellite instability-high cancer, gastric cancer, cervical cancer, hepatocellular cancer, Merkel cell cancer	NCT04335305
Anti-vascular endothelial growth factor	Bevacizumab	Binds circulating vascular endothelial growth factor	Colorectal cancer, non-squamous non-small cell lung cancer, glioblastoma, Renal cell cancer	NCT04305106, NCT04275414
Kinase inhibitor	Ruxolitinib	Inhibits Janus kinase (JAK) 1 and 2	Primary myelofibrosis, post-polycythemia vera myelofibrosis, and post-essential thrombocythemia myelofibrosis	NCT04338958, NCT04354714
	Imatinib	Inhibits bcr-abl tyrosine kinase	Chronic myeloid leukaemia, acute lymphoblastic leukaemia, gastrointestinal stromal tumours	NCT04357613, NCT04346147
	Acalabrutinib	Inhibits Bruton’s tyrosine kinase	Mantle cell lymphoma	NCT04346199
	Duvelisib	Inhibits phosphoinositide-3 kinase δ and γ	Chronic lymphocytic leukaemia, small lymphocytic lymphoma, follicular lymphoma	NCT04372602
Immunomodulator	Thalidomide	Immunomodulatory, antiangiogenic, and modulation of tumour necrosis factor-α	Multiple myeloma	NCT04273529, NCT04273581
	Lenalidomide	Immunomodulatory, antiangiogenic	Multiple myeloma	NCT04361643
Nuclear export inhibitor	Selinexor	Binds to exportin 1	Multiple myeloma	NCT04355676, NCT04349098
Granulocyte, macrophage-colony stimulating factor	Sargramostim	Haematopoietic growth factor and immune modulator	Shorten neutrophil recovery after induction chemotherapy	NCT04326920
Retinoid	Isotretinoin	Induces apoptosis	High-risk neuroblastoma	NCT04361422, NCT04353180
Interleukin	IL-2	Expansion and activation of regulatory T cells	Melanoma, renal cell cancer	NCT04357444
Cytotoxic chemotherapy	Etoposide	Topoisomerase II inhibitor	Testicular tumours, small cell lung cancer	NCT04356690
	Methotrexate	Antimetabolite, inhibits dihydrofolate reductase	Breast cancer, epidermoid cancers of the head and neck, cutaneous T cell lymphoma, squamous cell lung cancer, small cell lung cancer, non-Hodgkin’s lymphoma	NCT04352465
Radiotherapy	External beam radiation	DNA damage	Multiple cancer types	NCT04366791

A maximum of two representative trials have been included for a given agent.

Preliminary safety and efficacy data are currently available for only a few of these approved anticancer agents currently being tested in patients with COVID-19. In the CORIMUNO-19 trial, 129 patients with moderate or severe COVID-19 pneumonia received either tocilizumab plus standard treatment or standard treatment alone. The primary efficacy endpoint (a combination of the need for ventilation or death on day 14) was achieved in a significantly lower proportion of patients in the tocilizumab arm according to a pre-publication announcement.^14^ Preliminary data for 21 of the 25 patients treated with siltuximab in the SISCO trial showed that 76% of the patients had either stabilised or had demonstrated improved disease symptoms at the interim analysis.^15^ In an observational study of 2773 hospitalised COVID-19 patients, the in-hospital mortality among 786 patients who received systemic anticoagulation was 22.5% with a median survival of 21 days, compared with 22.8% and 14 days, respectively, in patients who did not receive anticoagulation.^16^ Eleven of the 31 patients in a retrospective review of patients with COVID‐19 had received corticosteroid treatment, and no association was observed between corticosteroid treatment and virus clearance time (hazard ratio [HR], 1.26; 95% confidence interval [CI], 0.58–2.74), hospital length of stay (HR, 0.77; 95% CI, 0.33–1.78) or duration of symptoms (HR, 0.86; 95% CI, 0.40–1.83).^17^ There are emerging and sometimes conflicting data regarding the use of corticosteroids in patients with COVID-19, including potential adverse effects on viral clearance and replication. Two patients who tested positive for the SARS-CoV-2 infection during the course of treatment with checkpoint inhibitors were reported to have recovered from the viral infection and will resume anticancer therapy.^18^

Anti-cytokines are among the most common classes of agents being tested for COVID-19. On the one hand, neutrophils and macrophages may secrete IL-6, TNF, IL-17A, granulocyte macrophage colony stimulating factor (CSF), and granulocyte CSF, all of which tip the scales in favour of hyperinflammation; on the other hand, regulatory T cells, natural killer cells, and B cells secrete IL-15, IFN-α, -β, and -γ, IL-12, and 1L-21, which aid viral clearance and hence need to be spared.^19^ There is, therefore, a need for caution in selecting which precise components of the cytokine system to target therapeutically in patients with COVID-19. For this reason, the National Cancer Institute has recently discouraged the use of JAK inhibitors in patients with COVID-19 since this class of agents has a broad anti-inflammatory action.^20^

### Conclusion

The COVID-19 pandemic has swiftly swept through the world, resulting in huge morbidity and significant mortality. Until an effective vaccine or antiviral specifically against SARS-CoV-2 is developed, there will remain a need for new and effective treatment for patients with severe COVID-19. Repurposed drugs targeting inflammation, immune dysfunction, and coagulopathy, including a variety of anticancer agents, should be evaluated systematically through well-designed and often novel trial platforms. The COVID-19 pandemic is an opportunity for the pharmaceutical and CRO industry, academia, and clinicians across a range of specialties to develop new models for the rapid evaluation of innovative therapeutic approaches.

## Data Availability

All data mentioned in this manuscript are from publicly available sources.
